# A Comparison of Performance of the Different Generations of Magnesium-Based Bioresorbable Coronary Stents

**DOI:** 10.3390/jcm15135003

**Published:** 2026-06-26

**Authors:** Jeremy Ang, Liam Marsden Back

**Affiliations:** 1 Cardiac Services, Townsville University Hospital, Townsville, QLD 4814, Australia; 2 Tasmanian School of Medicine, University of Tasmania, Hobart, TAS 7000, Australia; 3 Eastern Heart Clinic, Prince of Wales Hospital, Randwick, NSW 2031, Australia; liammarsdenback@gmail.com; 4 MRC Clinical Trials Unit, University College London, London WC1V 6LJ, UK

**Keywords:** bioresorbable, Magnesium, coronary, stent, Magmaris, Freesolve

## Abstract

**Background**: The permanence of contemporary coronary stents is associated with chronic complications, particularly in-stent restenosis and stent thrombosis. Poly-L-lactic-acid-based bioresorbable stents were withdrawn given late stent thrombosis risk. Magnesium-based bioresorbable stents (MgBRS) have emerged as promising alternatives. This study documents the safety and efficacy profile of MgBRS generations. **Methods**: A systematic review was performed using EMBASE, MEDLINE and Web of Science (2007 to August 2025). Studies include individuals requiring percutaneous coronary intervention for coronary artery disease with MgBRS. Primary outcomes were cardiac death, definite/probable scaffold thrombosis and target vessel failure (TVF), organised into ≤12, ≤24, ≤36 and ≤60 months. The secondary outcome was late lumen loss (LLL). **Results**: Four MgBRS generations were identified: AMS-1, DREAMS 1G, Magmaris and Freesolve; and 25 studies were included. Cardiac death and stent thrombosis rates were under 1.0% up to 24 months for all, and 0% with Freesolve at 36 months. AMS-1 had the highest TVF rate at 44.4% at 12 months, improving to 3.6% at 36 months in Freesolve. Only Magmaris had 60-month data, showing 3.4% cardiac death, 2.7% stent thrombosis and 16.4% TVF rates. The data suggest improvement in 12-month LLL: DREAMS 1G (0.4 ± 0.3 mm), Magmaris (0.5 ± 1.1 mm) and Freesolve (0.2 ± 0.4 mm). **Conclusions**: Each successive generation demonstrated improvement in all studied outcomes since the underperformance of AMS-1. The favourable performance of Freesolve is comparable to contemporary drug-eluting stents. Results of randomised head-to-head trials are anticipated.

## 1. Introduction

Interventional cardiology has evolved significantly since the first attempt at treating coronary artery disease in 1977 [1]. Initially, balloon angioplasty was developed to mechanically dilate stenotic regions of coronary arteries, but this carried risks of acute occlusion, elastic recoil, and vascular complications such as intimal dissections. To address these limitations, bare metal stents were introduced which provided the radial force necessary to counteract acute elastic recoil [2]. These stainless-steel scaffolds still have a high risk of in-stent thrombosis and in-stent restenosis due to their thrombogenicity and relatively low biocompatibility [3,4]. The risk of acute in-stent thrombosis was mitigated by the addition of single- and dual-antiplatelet therapy, at the expense of an increased risk of haemorrhagic complications [5].

Subsequent studies identifying neointimal hyperplasia as a major determinant of in-stent restenosis prompted the development of stents that were capable of releasing antiproliferative agents, thereby dampening vascular remodelling secondary to inflammation, proliferation and migration of vascular smooth muscle cells into the intima [6]. First- and second-generation DES consisted of a polymeric coating embedded with antiproliferative agents around a stent platform providing mechanical support [7]. The first-generation DES used sirolimus or paclitaxel antiproliferative drugs built around stainless-steel platforms; the second-generation DES platforms were changed to more biocompatible metallic alloys (e.g., cobalt-chromium or platinum-chromium) which also allowed for thinner struts and more flexibility and deliverability [8,9]. These polymers were essential not only as a drug carrier but also to modulate the controlled release of the drug over time. Their presence, however, has been implicated in the pathogenesis of short- and medium-term target vessel failure due to delayed epithelialisation and risk for stent thrombosis [10,11]. This prompted the development of third-generation DES characterised by biodegradable polymer coatings applied to metallic platforms enabling thinner struts, more flexibility and optimised drug release [12]. However, this generation has shown limited improvement in clinical outcomes over second-generation DES, limiting their adoption [13,14].

Despite their undoubted success in improving the treatment of obstructive coronary artery disease, these permanent implants still pose a risk of longer-term complications including in-stent restenosis, neoatherosclerosis and stent thrombosis [10,11,15,16]. As a proposed superior alternative, bioresorbable scaffolds were developed to degrade over time to address the temporary need for mechanical support while avoiding the late complications of permanent stents. Poly-L-lactic acid-based coronary scaffolds showed initial promise as a bioresorbable medium; however, they were associated with higher rates of stent-related complications [17], particularly stent thrombosis [18]. As an alternative design, Magnesium-based bioresorbable stents (MgBRS) have gained favour with regard to their bioresorbable profile and safety outcomes.

Four generations of MgBRS were identified and their characteristics are summarised in Table 1. The first-generation Absorbable Metal Stent (AMS-1, Biotronik AG, Bülach, Switzerland) MgBRS has the thickest strut and is the only MgBRS without an antiproliferative drug coating [19]. Since AMS-1, three versions of balloon-expandable Magnesium-based Drug-Eluting Absorbable Metal Scaffolds (DREAMS)) were produced: second-generation DREAMS 1G (Biotronik AG, Bülach, Switzerland), third-generation DREAMS 2G (commercial name “Magmaris”, Biotronik AG, Bülach, Switzerland), and fourth-generation DREAMS 3G (commercial name “Freesolve”, Biotronik AG, Bülach, Switzerland). This study is a systematic review and descriptive synthesis of the collected data which aims to compare the performance of the different generations of MgBRS with regard to key safety and efficacy outcomes.

## 2. Materials and Methods

This review was designed with the framework of the Preferred Reporting Items for Systematic Reviews and Meta-analyses (PRISMA) guidelines (Table S1). No patient or public involvement was required. All data collected was anonymous and no data protection issues were identified.

To be eligible for inclusion, a study must be published in English and have included individuals who required percutaneous coronary intervention for coronary artery disease and were subsequently treated with MgBRS. A publication was required to report the primary outcome of interest, able to be extracted as incidence rates on an intention-to-treat (ITT) basis. Studies were still included if the secondary outcome was not performed or unavailable.

The primary efficacy and safety outcomes collected were cardiac death, target vessel failure (TVF), and definite/probable scaffold thrombosis, which were organised into time periods of ≤12 months, ≤24 months, ≤36 months and ≤60 months. The secondary efficacy outcome of late lumen loss, as assessed by quantitative coronary angiography (QCA) or intravascular imaging, was collected where available, and converted to a standardised millimetre cross-sectional area for comparison.

A keyword systematic review was performed utilising databases EMBASE, MEDLINE and Web of Science, with date parameters from 2007 to August 2025. The year of 2007 was selected as this is the year of publication of the first in-human implantation of MgBRS.

The search utilised the following keywords

- Magnesium OR Mg OR Magmaris OR Freesolve.
- AND Bioresorbable OR Bioabsorbable OR Absorbable OR Dissolv*.
- AND Coronary (MeSH) and Stent (MeSH).
- AND Stent* OR Scaffold* OR Vascular scaffold* (MESH).

**Figure 1:**
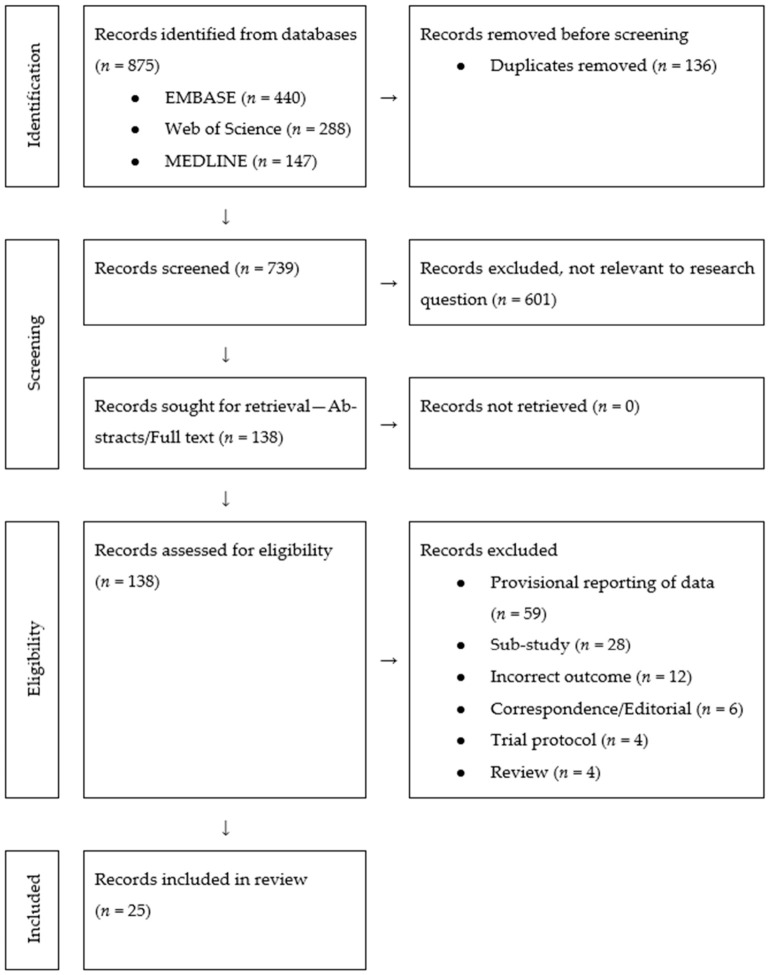
Magnesium-Based Bioresorbable Coronary Stents search strategy and study selection—completed using Preferred Reporting Items for Systematic Reviews and Meta-analyses flow diagram.

All clinical data, including single-arm observational studies, were included for screening. Restrictions of human-only trials, published from 2007 to present, were applied. Trade names of clinically studied MgBRS were included. Duplicate articles were removed and publications were screened by title and abstract for eligibility. Publications were subsequently retrieved for assessment. Reference lists of the included articles were also screened for missing publications. The details of the search strategy, screening and exclusions are summarised in Figure 1.

Data from included publications were pooled for analysis. Notably, discrepancies exist in the technical method of assessment for late lumen loss (LLL) measurements which are detailed in Table 2. All publications recorded LLL as the difference between the diameters at baseline and at follow-up in millimetres except for Fallesen et al. (a Magmaris study) which measured the difference between the areas at baseline and at follow-up in millimetre squared. To facilitate comparison, this was converted to diameters assuming a perfect circle and pooled with other Magmaris data in Table 3.

## 3. Results

The combined searches identified 875 records, with 739 remaining after removing duplicates. After initial screening, a further 601 were excluded following the review of the title and abstract. Of the remaining 138 studies, 113 were excluded. Twenty-five studies involving the use of MgBRS were eligible for inclusion into this study: one study involving 63 patients for AMS-1; one study involving 46 patients for DREAMS 1G; 21 studies totalling 3582 patients for Magmaris; and two studies with 116 patients for Freesolve. All of the individual studies for AMS-1, DREAMS 1G and Freesolve were prospective single-arm trials; and of the 21 Magmaris studies, three were randomised controlled trials, one was a retrospective observational single-arm study and the remaining 17 were prospective observational single-arm studies. The studies are summarised in Table S2.

Furthermore, AMS-1, DREAMS 1G and Freesolve publications included only de novo coronary artery lesions. At least 30% of Magmaris interventions and 79% with Freesolve were graded to be ACC/AHA Lesion Classification B2/C. In the MgBRS trials, 20 of 25 reported on the use of intravascular imaging, with a range of 8.8–100%; however, a majority reported >50% use. Importantly, the BIOMAG-1 study, the only study investigating the 4th-generation Freesolve MgBRS, reports a 97% penetrance of intravascular imaging use. Patient, lesion and procedural characteristics of the included MgBRS trials have been included in Table S3.

### 3.1. Risk of Bias Assessment

Risk of Bias assessment was made using the Cochrane Risk of Bias tool for randomised studies (RoB 2), and Cochrane Risk of Bias tool for non-randomised studies (ROBINS-1) [20,21]. A summary of these findings is provided in Table 4. The Risk of Bias in non-randomised trials of MgBRS was variable given the heterogeneity of trial designs. Multiple studies allowed proceduralists select patients for MgBRS implantation by utilising clinical experience and without specific criteria to guide appropriateness for inclusion. Although crossover to conventional stenting with DES during the index MgBRS procedure was relatively rare, some trials reported a disproportionately high rate (such as >25% in BIFSORB PILOT-II). Lastly, while clinical follow-up was excellent across the majority of trials (Table S4), the MAGMARIS ACS REGISTRY, BIFSORB PILOT-II and IT MASTERS REGISTRY reported follow-up rates below 80%.

### 3.2. Primary and Secondary Outcomes

#### 3.2.1. Cardiac Death

Rates of cardiac death remained low across all four generations. Patients receiving AMS-1 and DREAMS 1G MgBRS had no recorded cardiac deaths at the 12-month follow-up. Magmaris was the only stent with a 60-month follow-up, showing a low risk of cardiac death at 0.6% at 24 months which increased to 3.4% at 60 months. To date, fourth-generation Freesolve has 0% cardiac death risk at 36 months. Cardiac death outcomes are shown in Table 5.

#### 3.2.2. Target Vessel Failure

The original MgBRS design AMS-1 performed poorly with regard to TVF, showing a 44.4% TVF rate at 12 months. With the introduction of drug-eluting design, the TVF rates of MgBRS designs dropped to 7.0% with DREAMS 1G at 12 months and further declined to 3.6% with Freesolve at 36 months. Once again, Magmaris was the only MgBRS with 60-month data, showing a 7.3% and 16.4% TVF rates at 24 and 60 months respectively. TVF outcomes are shown in Table 6.

#### 3.2.3. Definite or Probable Stent Thrombosis

Stent thrombosis remained low in all MgBRS groups. There were no recorded cases of definite or probable stent thrombosis at 12 months with AMS-1 and DREAMS 1G. At 12 months, Magmaris had a 0.8% stent thrombosis risk which remained low at 2.7% during the 60-month follow-up. Thus far, Freesolve has demonstrated excellent performance with no cases of stent thrombosis at 36 months. Define or probable stent thrombosis outcomes are shown in Table 7.

#### 3.2.4. Late Lumen Loss

Late lumen loss outcome data was not reported in AMS-1 and only five of the 21 Magmaris studies evaluated LLL. Only 12-month follow-up data were available. Here, the data suggests an improvement in late lumen loss at 12 months with each generation: DREAMS 1G (0.4 ± 0.3 mm), Magmaris (0.5 ± 1.1 mm) and Freesolve (0.2 ± 0.4 mm). LLL outcomes are shown in Table 3.

## 4. Discussion

Despite only being tested in a small trial, the performance of the first generation MgBRS was significantly inferior compared to its subsequent MgBRS generations, especially with regard to rates of target vessel failure. Without any drug-eluting properties and a thicker strut, particularly when compared to contemporary generation DES of everolimus-eluting stents and zotarolimus-eluting stents, in retrospect, the suboptimal performance of AMS-1 is not unexpected [55,56]. With better design which included thinner struts and drug-elution, the third and fourth MgBRS generations have demonstrated favourable 12- and 24-month clinical outcomes.

In the wake of the suboptimal performance of PLLA bioresorbable coronary stents [17], stent thrombosis should be reviewed with particular importance for bioresorbable scaffolds. This complication is theorised to arise from the thrombogenic foreign material of the coronary scaffolds prior to endothelialisation [11]. For example, Yamaji et al. founded that the leading mechanism of very late stent thrombosis in PLLA bioresorbable coronary stents were from scaffold discontinuity which may suggest an unfavourable resorption profile, followed by device malapposition and neoatherosclerosis [57]. With current MgBRS devices, endothelialisation progresses as the scaffold resorbs, such that complete or near-complete resorption will have occurred by 12 months following implantation [58]. For instance, over 99% of patients with fourth-generation Freesolve MgBRS showed homogenous resorption at 12 months using optical coherence tomography [53]. The risk of very late stent thrombosis should, thus, quiesce following complete resorption of the polymer, contrasting with the chronic risk of permanent metallic scaffolds. Notably, Freesolve appears to have significantly outperformed its predecessor Magmaris in this regard, as demonstrated in in vivo and in vitro studies. In addition, the Magnesium polymer is theorised to have native antithrombotic properties which may aid in its superior performance. A novel in vitro experiment by Müller et al. measured the thrombogenicity of MgBRS using platelet-containing plasma from the same healthy human donor. In this experiment, Freesolve exhibits significantly less irreversible platelet adhesion, in keeping with the findings of the BIOMAG-1 trial [59]. Interestingly, the observed rise in percentage of definite or probable stent thrombosis following resorption in Magmaris MgBRS at 36 and 60 months may be due to follow-up bias, with a disproportionate decline in the total number of patients followed-up compared to those with probable or definite stent thrombosis. This is further suggested by the similar absolute number of recorded cases at 24 months following implantation (i.e., 12 months following resorption) from a 12-month follow-up. Other possible mechanisms may include edge dissections, residual un-resorbed fragments or irregular resorption patterns [60,61,62]. Further research is required to guide the recommended duration of dual antiplatelet therapy following complete MgBRS resorption in patients without other significant coronary artery disease.

At present, the evidence surrounding long-term intravascular imaging outcomes in patients who have been treated with MgBRS remains limited. While MgBRS presents a promising alternative to permanent coronary stents due to its ability to gradually resorb and thus avoid permanent foreign material in the vessel, the exact effects on vascular healing and remodelling remain incompletely understood. Available imaging studies suggest at least partial LLL compared to baseline. Early on, paclitaxel-coated DREAMS 1G had a higher LLL than contemporary DES; however, a switch to limus-derived antiproliferative MgBRS and better scaffold design (e.g., thinner struts) has resulted in comparable LLL in Freesolve MgBRS [63,64]. The vascular response after completed scaffold bioresorption may include significant LLL and vascular remodelling, which is critical to understanding the performance of MgBRS and guiding future use. As such, longitudinal studies should consider incorporating routine angiographic and intravascular evaluation beyond 36 months to improve our understanding of vascular remodelling following scaffold absorption [65], and ultimately inform clinical decision making in the management of coronary syndromes requiring percutaneous coronary intervention.

### Limitations

Given the recency of the inception of MgBRS technology, a significant number of publications analysed were single-arm trials. As described, this includes all AMS-1, DREAMS 1G and Freesolve individual publications, and 18 of 21 Magmaris publications (17 prospective, one retrospective). In most of these, patients were included into the MgBRS arm based on the discretion of the operator which may introduce selection bias. Of those Magmaris studies that reported lesion complexity, however, coronary artery lesions which were classified as ACC/AHA B2 or C lesions accounted for at least 30% of cases. Of note, most of Freesolve recipients (approximately 77%) were classified as ACC/AHA B2 or C lesions.

The use of intravascular imaging (either OCT or IVUS) varies greatly across studies, ranging from 7% to 100% use. Intravascular imaging was not used or reported in 5 studies—all of which were Magmaris studies. This may confound the interpretation of the clinical outcomes as their success is significantly influenced by intravascular imaging guidance. However, due to the heterogeneity of data and limitations in reporting between studies, a subgroup analysis stratifying clinical outcomes based on high or low use of intravascular imaging could not be performed. Of note, most contemporary trials mandate the use of intravascular imaging, and BIOMAG-1, a 2023 Freesolve study, reported a 97% rate of intravascular imaging use.

Pooling and comparing data with regard to LLL was difficult as there was significant paucity of follow-up data, and, where available, differing methods were employed to objectively measure the degree of late lumen loss. To facilitate comparison between the studies, LLL measurements in Fallesen et al.’s study which calculated the difference in the minimal areas at baseline and at follow-up (in mm^2^) were converted to difference in the diameters as measured by all remaining publications. This geometrical conversion (detailed in Equation (S1) and Table S5) makes the assumption that the cross-sectional areas represent a perfect circle and that orthogonal measurements to the coronary arteries were made. This methodological choice provides an exploratory analysis with some abstract values for comparison between studies; however, more robust prospective studies with prespecified imaging protocols are required to confidently understand the relationship of lumen loss to MgBRS. Future studies should also aim to perform intravascular imaging as a default with standardised measurement protocols clearly defined in their study protocols.

While Magmaris was evaluated in the greatest number of studies, there was a significant reduction in patient outcomes measured at 36 and 60 months with only 486 and 146 patients outcomes respectively. This relative drop in available patient outcome data was predominantly contributed by the BIOSOLVE IV study which provided the highest number of patient outcomes up to 24 months only. This may have implications to the accuracy and generalisability of this review, particularly with regard to Magmaris MgBRS after 24 months. Furthermore, as Freesolve was only evaluated in a significantly smaller number of patients compared to Magmaris, broad conclusions directly comparing the efficacy between these two devices cannot be drawn.

## 5. Conclusions

MgBRS technology has the potential for providing temporary vascular support and facilitating revascularisation while gradually degrading after fulfilling their immediate scaffolding function. This avoids the longer-term complications of permanent stents including in-stent restenosis, neoatherosclerosis and stent thrombosis, in addition to being able to allow all future options for coronary intervention. This systematic review has demonstrated notable performance improvements with each subsequent MgBRS generation; however, there is limited long-term data beyond 24 months. Large randomised control trials with good short- and long-term follow-up, along with planned late angiographic assessment to evaluate vascular response following bioresorption of MgBRS are necessary before any recommendations for general use can be made. The results of the randomised BIOMAG-II study comparing MgBRS to contemporary DES are eagerly awaited to guide future use (ClinicalTrials.gov identifier: NCT05540223) [66]. With continual advancement in bioresorbable stent technologies, MgBRS has the potential to vastly improve options for contemporary treatment of obstructive coronary artery disease.

## Figures and Tables

**Table 1:** MgBRS characteristics from included trials.

Stent Name	First Publication	Manufacturer	Deployment	Material	Antiproliferative Drug	Approximate Resorption Time (Months)	Available Sizes (mm)	Strut Thickness (µm)	Relative Struct Thickness
AMS-1	2007	Biotronik	Balloon expandable	Mg stent	None	1	Ø 3.0, 3.5 Length: 10, 15	165	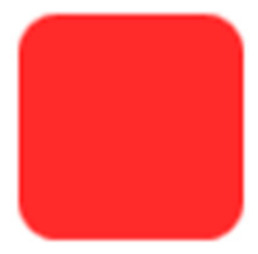
DREAMS 1G	2013	Biotronik	Balloon expandable	Mg stent, PLGA coating	Paclitaxel	9–12	Ø 3.25, 3.5 Length: 15	120	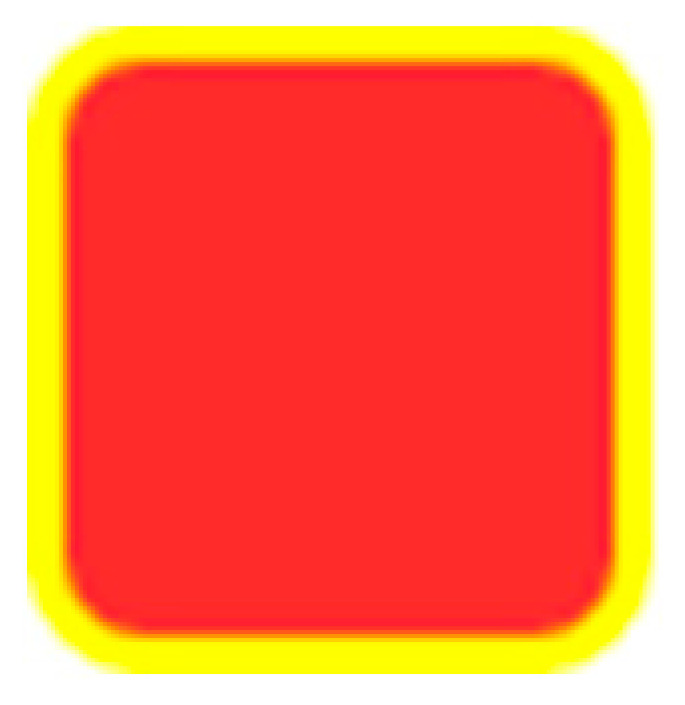
‘Magmaris”—DREAMS 2G	2016	Biotronik	Balloon expandable	Mg stent, PLLA coating	Sirolimus	12	Ø 2.5, 3.0, 3.5 Length: 15, 20, 25	150	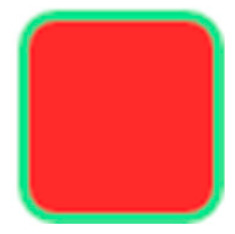
“Freesolve”—DREAMS 3G	2023	Biotronik	Balloon expandable	Mg stent, PLLA coating	Sirolimus	12	Ø 2.5, 3.0, 3.5, 4.0 Length: 13, 18, 22, 26, 30	99	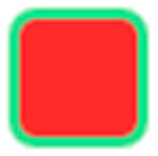

The cross-sectional area of each device relative to each other is represented in red, with strut thickness represented either in yellow (paclitaxel) or green (sirolimus). Mg, Magnesium; Ø, diameter; PLGA, poly-lactic-co-glycolic acid; PLLA, poly-L-lactic acid.

**Table 2:** Characteristics of publications that measured late lumen loss at 12 months.

Study Name	Qualitative Method	Follow-Up	Late Lumen Loss
BIOSOLVE-1	QCA/IVUS	73.9%	0.4 ± 0.3 mm 1.3 ± 0.7 mm^2^ (MLA IVUS-guided)
MAGSTEMI	QCA	87.8%	0.4 ± 0.5 mm
Guitierrez-Barrios et al.	QCA	36.7%	0.6 ± 0.8 mm
PRAGUE-22	QCA/OCT	60%	0.5 ± 0.7 mm (QCA) 0.6 ± 0.4 mm (OCT)
Fallesen et al.	OCT	84%	2.3 ± 1.6 mm^2^ (MLA OCT-guided) 1.4 ± 1.4 mm^2^ (MLA Angiography-guided)
BIOMAG-1	QCA	86.0%	0.2 ± 0.4 mm

QCA, quantitative coronary angiography; IVUS, intravascular ultrasonography; MLA, minimal luminal area; OCT, optical coherence tomography.

**Table 3:** Pooled late lumen loss outcomes from included trials, standardised to diameters.

Stent Name	≤12 Months (mm)
AMS-1	NA
DREAMS 1G	0.4 ± 0.3
MAGMARIS	0.5 ± 1.1
FREESOLVE	0.2 ± 0.4

NA, not available.

**Table 4:** Risk of Bias assessment for included Magnesium-based Bioresorbable Coronary Stents.

	Randomisation Process	Selection of Participants	Classification of Interventions	Deviations from Intended Interventions	Missing Outcome Data	Outcome Measurement	Selection of the Reported Result	Overall Bias
PROGRESS-AMS [22]		Some	Low	Some	Low	Low	Low	Some
BIOSOLVE-I [23,24]		Low	Low	Low	Low	Low	Low	Low
BIOSOLVE-II [25,26,27,28]		Low	Low	Low	Low	Low	Low	Low
BIOSOLVE-III [29,30]		Low	Low	Low	Low	Low	Low	Low
BEST-MAG [31]		Low	Low	Low	Low	Low	Low	Low
MAGMARIS ACS REGISTRY [32,33]		Low	Low	Low	Some	Low	Low	Some
MAGSTEMI [34,35]	Low	Low	Low	Low	Low	Low	Low	Low
Ghafari et al. [36]		Low	Low	Low	Low	Low	Low	Low
Carlier et al. [37]		Some	Low	Low	Low	Low	Low	Some
BIOSOLVE-IV [38,39]		Low	Low	Low	Low	Low	Low	Low
CardioHULA REGISTRY [40]		Low	Low	Low	Low	Low	Low	Low
Franze et al. [41]		Some	Low	Low	Low	Low	Low	Some
Guitierrez-Barrios et al. [42]		Low	Low	Low	Low	Low	Low	Low
BIFSORB Pilot II [43]		Some	Low	Some	Some	Low	Low	Some
PRAGUE-22 [44]	Low	Low	Low	Low	Low	Low	Some	Some
Fallesen et al. [45]	Low	Some	Low	Low	Low	Low	Low	Some
MULTICENTRE ITALIAN REGISTRY [46]		Low	Low	Low	Low	Low	Low	Some
Al Nooryani et al. [47]		Some	Low	Low	Low	Low	Low	Some
Bossard et al. [48]		Some	Low	Low	Low	Low	Low	Some
Truong et al. [49]		Low	Low	Low	Low	Low	Low	Low
INTERNATIONAL MULTICENTRE DISCO REGISTRY [50]		Low	Low	Low	Low	Low	Low	Low
SHERPA MAGIC STUDY [51]		Low	Low	Low	Low	Low	Low	Low
IT MASTERS REGISTRY [52]		Low	Low	Low	Some	Low	Low	Some
BIOMAG-1 [53,54]		Low	Low	Low	Low	Low	Low	Low

**Table 5:** Pooled cardiac death outcomes from included trials.

Stent Name	≤12 Months	≤24 Months	≤36 Months	≤60 Months
AMS-1	0/63 (0%)	NA	NA	NA
DREAMS 1G	0/43 (0%)	NA	NA	NA
MAGMARIS	12/3198 (0.4%)	18/2813 (0.6%)	10/486 (2.1%)	5/146 (3.4%)
FREESOLVE	0/114 (0%)	0/115 (0%)	0/112 (0%)	NA

NA, not available.

**Table 6:** Pooled target vessel failure outcomes from included trials.

Stent Name	≤12 Months	≤24 Months	≤36 Months	≤60 Months
AMS-1	28/63 (44.4%)	NA	NA	NA
DREAMS 1G	3/43 (7.0%)	NA	NA	NA
MAGMARIS	177/3198 (5.5%)	205/2813 (7.3%)	50/486 (10.3%)	24/146 (16.4%)
FREESOLVE	4/116 (3.5%)	4/115 (3.5%)	4/112 (3.6%)	NA

NA, not available.

**Table 7:** Pooled definite or probable stent thrombosis outcomes from included trials.

Stent Name	≤12 Months	≤24 Months	≤36 Months	≤60 Months
AMS-1	0/63 (0%)	NA	NA	NA
DREAMS 1G	0/43 (0%)	NA	NA	NA
MAGMARIS	25/3193 (0.8%)	22/2813 (0.8%)	8/486 (1.7%)	4/146 (2.7%)
FREESOLVE	0/114 (0%)	0/115 (0%)	0/112 (0%)	NA

NA., not available.

## Supplementary Materials

The following supporting information can be downloaded at: https://www.mdpi.com/article/10.3390/jcm15135003/s1, Table S1: PRISMA 2020 Checklist. Table S2: Summary of Magnesium-Based Bioresorbable Coronary Stent Trials presented in chronological order of publication. Table S3: Patient, Lesion and Procedure Characteristics from Magnesium-Based Bioresorbable Coronary Stent Trials. Table S4: Clinical Follow-Up Rates from Included Magnesium-Based Bioresorbable Stent Trials. Equation (S1): Equation used to convert area to diameter. Table S5: Optical coherence tomography and quantitative angiography data from Fallesen et al. on late lumen loss.

## Abbreviations

The following abbreviations are used in this manuscript:

ACC	American College of Cardiology
AHA	American Heart Association
DES	Drug-eluting stents
DREAMS	Drug-Eluting Absorbable Metal Scaffold
ITT	Intention to treat
IVUS	Intravascular ultrasonography
LLL	Late lumen loss
MgBRS	Magnesium-based bioresorbable stents
MLA	Minimal luminal area
NA	Not available
OCT	Optical coherence tomography
PLGA	Poly-lactic-co-glycolic acid
PLLA	Poly-L-lactic acid
PRISMA	Preferred Reporting Items for Systematic Reviews and Metaanalyses
QCA	Quantitative coronary angiography
TVF	Target vessel failure
