# Supplementary material for: A Comparison of Performance of the Different Generations of Magnesium-Based Bioresorbable Coronary Stents
Source: J Clin Med. 2026 Jun 26;15(13):5003. doi: 10.3390/jcm15135003 (PMC13361727; doi:10.3390/jcm15135003)
Supplement: Supplementary file 1 [file jcm-15-05003-s001.zip › jcm-4341154-supplementary.pdf]

PRISMA

PRISMA 2020 Checklist

Table S1. PRISMA 2020 Checklist.

Section and Topic	Item #	Checklist item	Location where item is reported
**TITLE**			
Title	1	Identify the report as a systematic review.	1, 2
**ABSTRACT**			
Abstract	2	See the PRISMA 2020 for Abstracts checklist.	1
**INTRODUCTION**			
Rationale	3	Describe the rationale for the review in the context of existing knowledge.	1, 2
Objectives	4	Provide an explicit statement of the objective(s) or question(s) the review addresses.	2
**METHODS**			
Eligibility criteria	5	Specify the inclusion and exclusion criteria for the review and how studies were grouped for the syntheses.	3
Information sources	6	Specify all databases, registers, websites, organisations, reference lists and other sources searched or consulted to identify studies. Specify the date when each source was last searched or consulted.	3
Search strategy	7	Present the full search strategies for all databases, registers and websites, including any filters and limits used.	3, 4
Selection process	8	Specify the methods used to decide whether a study met the inclusion criteria of the review, including how many reviewers screened each record and each report retrieved, whether they worked independently, and if applicable, details of automation tools used in the process.	3, 4
Data collection process	9	Specify the methods used to collect data from reports, including how many reviewers collected data from each report, whether they worked independently, any processes for obtaining or confirming data from study investigators, and if applicable, details of automation tools used in the process.	3, 4
Data items	10a	List and define all outcomes for which data were sought. Specify whether all results that were compatible with each outcome domain in each study were sought (e.g. for all measures, time points, analyses), and if not, the methods used to decide which results to collect.	3
	10b	List and define all other variables for which data were sought (e.g. participant and intervention characteristics, funding sources). Describe any	4,

Section and Topic	Item #	Checklist item	Location where item is reported
		assumptions made about any missing or unclear information.	Supplement
Study risk of bias assessment	11	Specify the methods used to assess risk of bias in the included studies, including details of the tool(s) used, how many reviewers assessed each study and whether they worked independently, and if applicable, details of automation tools used in the process.	5-7
Effect measures	12	Specify for each outcome the effect measure(s) (e.g. risk ratio, mean difference) used in the synthesis or presentation of results.	3, 4
Synthesis methods	13a	Describe the processes used to decide which studies were eligible for each synthesis (e.g. tabulating the study intervention characteristics and comparing against the planned groups for each synthesis (item #5)).	Supplement
	13b	Describe any methods required to prepare the data for presentation or synthesis, such as handling of missing summary statistics, or data conversions.	4, 5
	13c	Describe any methods used to tabulate or visually display results of individual studies and syntheses.	Supplement
	13d	Describe any methods used to synthesize results and provide a rationale for the choice(s). If meta-analysis was performed, describe the model(s), method(s) to identify the presence and extent of statistical heterogeneity, and software package(s) used.	3-4
	13e	Describe any methods used to explore possible causes of heterogeneity among study results (e.g. subgroup analysis, meta-regression).	NA
	13f	Describe any sensitivity analyses conducted to assess robustness of the synthesized results.	NA
Reporting bias assessment	14	Describe any methods used to assess risk of bias due to missing results in a synthesis (arising from reporting biases).	5-7
Certainty assessment	15	Describe any methods used to assess certainty (or confidence) in the body of evidence for an outcome.	NA
**RESULTS**			
Study selection	16a	Describe the results of the search and selection process, from the number of records identified in the search to the number of studies included in the review, ideally using a flow diagram.	4
	16b	Cite studies that might appear to meet the inclusion criteria, but which were excluded, and explain why they were excluded.	NA

PRISMA 2020 Checklist

Section and Topic	Item #	Checklist item	Location where item is reported
Study characteristics	17	Cite each included study and present its characteristics.	Supplement
Risk of bias in studies	18	Present assessments of risk of bias for each included study.	5-7
Results of individual studies	19	For all outcomes, present, for each study: (a) summary statistics for each group (where appropriate) and (b) an effect estimate and its precision (e.g. confidence/credible interval), ideally using structured tables or plots.	Supplement
Results of syntheses	20a	For each synthesis, briefly summarise the characteristics and risk of bias among contributing studies.	5-7, supplement
	20b	Present results of all statistical syntheses conducted. If meta-analysis was done, present for each the summary estimate and its precision (e.g. confidence/credible interval) and measures of statistical heterogeneity. If comparing groups, describe the direction of the effect.	7-8
	20c	Present results of all investigations of possible causes of heterogeneity among study results.	NA
	20d	Present results of all sensitivity analyses conducted to assess the robustness of the synthesized results.	NA
Reporting biases	21	Present assessments of risk of bias due to missing results (arising from reporting biases) for each synthesis assessed.	5-7
Certainty of evidence	22	Present assessments of certainty (or confidence) in the body of evidence for each outcome assessed.	NA
**DISCUSSION**			
Discussion	23a	Provide a general interpretation of the results in the context of other evidence.	7-10
	23b	Discuss any limitations of the evidence included in the review.	9, 10
	23c	Discuss any limitations of the review processes used.	9, 10
	23d	Discuss implications of the results for practice, policy, and future research.	9, 10
**OTHER INFORMATION**			

PRISMA

PRISMA 2020 Checklist

Section and Topic	Item #	Checklist item	Location where item is reported
Registration and protocol	24a	Provide registration information for the review, including register name and registration number, or state that the review was not registered.	NA
	24b	Indicate where the review protocol can be accessed, or state that a protocol was not prepared.	NA
	24c	Describe and explain any amendments to information provided at registration or in the protocol.	NA
Support	25	Describe sources of financial or non-financial support for the review, and the role of the funders or sponsors in the review.	11
Competing interests	26	Declare any competing interests of review authors.	11
Availability of data, code and other materials	27	Report which of the following are publicly available and where they can be found: template data collection forms; data extracted from included studies; data used for all analyses; analytic code; any other materials used in the review.	11

From: Page MJ, McKenzie JE, Bossuyt PM, Boutron I, Hoffmann TC, Mulrow CD, et al. The PRISMA 2020 statement: an updated guideline for reporting systematic reviews. BMJ 2021;372:n71. doi: 10.1136/bmj.n71.

This work is licensed under CC BY 4.0. To view a copy of this license, visit https://creativecommons.org/licenses/by/4.0/

Table S2. Summary of Magnesium-Based Bioresorbable Coronary Stent Trials presented in chronological order of publication.

Study Name	Year First Published	Study Type	Centres	Device Type	Comparator Arm	Patients, *n*	Patients, *n*	Available Outcome Data		Follow-Up Duration (Months)
						MgBRS	DES	Primary Safety/Efficacy	Secondary Efficacy	
PROGRESS-AMS [22]	2007	Single arm, prospective	8	AMS	NA	63	NA	Y	N	4
BIOSOLVE-I [23,24]	2013	Single arm, prospective	5	DREAMS 1G	NA	46	NA	Y	Y	1, 6, 12, 24, 36
BIOSOLVE-II [25–28]	2016	Single arm, prospective	13	MAGMARIS	NA	123	NA	Y	Y–25 patients	6, 12, 36, 60

BIOSOLVE-III [29,30]	2018	Single arm, prospective, observational	13	MAGMARIS	NA	61 (+123 Biosolve II)	NA	Y	N	12, 36
BEST-MAG [31]	2018	Single arm, prospective, observational	1	MAGMARIS	NA	30	NA	Y	N	12
MAGMARIS ACS REGISTRY [32,33]	2018	Single arm, prospective, observational	1	MAGMARIS	NA	193	NA	Y	N	1, 12, 24.
MAGSTEMI [34,35]	2019	RCT	11	MAGMARIS	SES	74	76	Y	Y	12, 36
Ghafari et al. [36]	2019	Single arm, prospective, observational	1	MAGMARIS	NA	29	NA	Y	N	6
Carlier et al. [37]	2020	Single arm, prospective, observational	1	MAGMARIS	NA	35	NA	Y	N	6
BIOSOLVE-IV [38,39]	2020	Single arm, prospective	80	MAGMARIS	NA	1075	NA	Y	N	12, 24, 36
CardioHULA REGISTRY [40]	2020	Single arm, prospective, observational	1	MAGMARIS	NA	42	NA	Y	N	12
Franze et al. [41]	2021	Single arm, prospective, observational	10	MAGMARIS	NA	175	NA	Y	N	12
Guitierrez-Barrios et al. [42]	2021	Single arm, prospective, observational	5	MAGMARIS	NA	90	NA	Y	Y	6, 12, 24
BIFSORB Pilot II [43]	2021	Single arm, prospective, observational, proof-of-concept.	1	MAGMARIS	NA	20	NA	Y	N	1

PRAGUE-22 [44]	2021	RCT	2	MAGMARIS	DES	25	25	Y	Y	12
Fallesen et al. [45]	2022	RCT	1	MAGMARIS (OCT-guided implantation)	MAGMARIS (Angiography-guided implantation	75	NA	Y	Y	6
MULTICENTRE ITALIAN REGISTRY [46]	2022	Single arm, prospective, observational	4	MAGMARIS	NA	207	NA	Y	N	12, 24
Al Nooryani et al. [47]	2022	Single arm, prospective, observational	1	MAGMARIS	NA	54	NA	Y	N	30
Bossard et al. [48]	2022	Single arm, prospective, observational	1	MAGMARIS	NA	84	NA	Y	N	60
Truong et al. [49]	2023	Single arm, prospective, observational	1	MAGMARIS	NA	60	NA	Y	N	12
INTERNATIONAL MULTICENTRE DISCO REGISTRY [50]	2023	Single arm, retrospective cohort	26	MAGMARIS	NA	12	NA	Y	N	6
SHERPA MAGIC STUDY [51]	2023	Single arm, prospective, observational	18	MAGMARIS	NA	543	NA	Y	N	12
IT MASTERS REGISTRY [52]	2023	Single arm, prospective, observational	14	MAGMARIS	NA	350	NA	Y	N	12
BIOMAG-1 [53,54]	2023	Single arm, prospective	14	FREESOLVE	NA	116	NA	Y	N	6, 12, 36

MgBRS, Magnesium-based bioresorbable stents; DES, drug-eluting stent; NA, not applicable, Y, reported, N, not reported.

Table S3. Patient, Lesion and Procedure Characteristics from Magnesium-Based Bioresorbable Coronary Stent Trials.

	Patient Characteristics			Inclusion Criteria				Exclusion Criteria							
**Study Name**	**Coronary Syndrome**	**Age (mean +/− SD)**	**Male (%)**	**De Novo Lesion**	**Reference Vessel Diameter (mm)**	**Lesion Length (mm)**	**Lesion Stenosis**	**LVEF < 30%/Cardiogenic Shock**	**Severe Calcification**	**Left Main**	**Bifurcation**	**Grafted Vessel**	**ACC/AHA Lesion Classification B2/C (%)**	**Intravascular Imaging, %**	**Antiplatelet Regimen**
PROGRESS-AMS [22]	X	61.3 +/− 9.5	70%	De Novo	X	X	X	X	X	X	X	X	X	IVUS, 100%	X
BIOSOLVE-I [23,24]	Stable or unstable angina, or silent ischaemia.	65.3 +/− 9.7	73.9 %	De Novo	3–3.5	<12	50–99%	Excluded	Excluded	Excluded	Excluded	Excluded	X	OCT/IVUS, 93.5%	DAPT 12 months
BIOSOLVE-II [25–28]	Stable or unstable angina, or silent ischaemia.	65.2 +/− 10.3	63%	De Novo	2.2–3.7	<21	50–99%	Excluded	Excluded	Excluded	Excluded	Excluded	44%	OCT/IVUS, 24.3%	DAPT 6 months
BIOSOLVE-III [29,30]	Stable or unstable angina, or silent ischaemia	65.5 +/− 10.8	63%	De Novo	2.7–3.8	<21	50–99%	Excluded	Excluded	Excluded	Excluded	Excluded	61%	X	DAPT 6 months
BEST-MAG [31]	STEMI	54.7 +/− 10.2	76.7 %	All lesions	2.7–4.0	<21	X	Excluded	Excluded	Excluded	Excluded	X	X	OCT/IVUS, 17%	Aspirin/Ticagrelor 12 months
MAGMARIS ACS REGISTRY [32,33]	Acute coronary syndrome including STEMI	66.3 +/− 8.9	77.7 %	All lesions	2.7–3.7	<21	50–99%	Included	Excluded	Included	Included	X	26.6%	OCT, 20%	DAPT 12 months
MAGSTEMI [34,35]	STEMI	58.8 +/− 10.6	85.1 %	De Novo	2.75–3.75	All lesions	X	Included	Excluded	Included	Included	X	X	X	DAPT 12 months
Ghafari et al. [36]	All coronary presentations	54 +/− 60	75%	De Novo	X	X	X	X	X	X	X	X	X	OCT/IVUS, 8.8%	X

	at clinicians' discretion														
Carlier et al. [37]	All coronary syndromes at clinicians' discretion	55 +/− 7	77%	De Novo	X	X	X	X	X	X	X	X	X	X	X
BIOSOLVE-IV [38,39]	Symptomatic coronary disease excluding STEMI	61.3 +/− 10.5	75%	De Novo	2.7–3.7	<21	50–99%	Excluded	Included	Excluded	Excluded	Excluded	15.2%	X	DAPT 6 months
CardioHULA REGISTRY [40]	All coronary syndromes at clinicians' discretion	58.9 +/− 11	85.7 %	De Novo	2.5–3.5	All lesions	X	Excluded	Excluded	Excluded	Excluded	Excluded	X	OCT, 100%	DAPT, guideline per presentation
Franze et al. [41]	All coronary syndromes at clinicians' discretion	55.5 +/− 9	85.1 %	De Novo	X	X	X	Excluded	Included	Included	Included	X	X	OCT/IVUS, 61.7%	X
Guitierrez-Barrios et al. [42]	Acute coronary syndrome including STEMI	55.9 +/− 9.9	75.6 %	De Novo	3.0–3.5	<20	X	Excluded	Excluded	Excluded	Excluded	X	X	OCT, 68.9%	DAPT 12 months
BIFSORB Pilot II [43]	Stable angina, NSTEMI, or silent ischaemia	66 +/− 10	82%	De Novo bifurcation lesion	X	X	X	Excluded	Excluded	Excluded	Included	X	X	OCT, 100%	DAPT, duration not reported
PRAGUE-22 [44]	Acute Coronary Syndrome including STEMI	57 +/− 10.5	64%	De Novo	2.7–3.7	All lesions	X	Excluded	Excluded	Excluded	X	X	X	OCT, 92%	DAPT 12 months
Fallesen et al. [45]	NSTEMI	61.1 +/− 10.9	79.9 %	De Novo	2.75–4.0	<28	>50%	Included	Included	Included	Included	Included	44%	OCT, 50%	Aspirin/Ticagrelor 12 months
MULTICENTRE ITALIAN REGISTRY [46]	All coronary presentations at clinicians' discretion	60.8 +/− 9.7	83%	All lesions	All lesions	All lesions	X	Included	Recommended to avoid	Recommended to avoid	Excluded	Recommended to avoid	X	OCT/IVUS, 47%	DAPT 6 months minimum

Al Nooryani et al. [47]	All coronary presentations at clinicians' discretion	54 +/− 11	85%	All lesions	All lesions	All lesions	X	Excluded	Included	Included	Included	Included	X	OCT/IVUS, 80%	DAPT 12 months
Bossard et al. [48]	All coronary presentations at clinicians' discretion	62 +/− 11	75%	De Novo	All lesions	All lesions	X	X	Included	Included	Included	Included	35%	OCT/IVUS, 7%	DAPT 12 months
Truong et al. [49]	All coronary presentations at clinicians' discretion	59.4 +/− 10.4	71%	All lesions	<3.75	All lesions	X	Excluded	Excluded	Excluded	X	X	45%	IVUS, 98.3%	DAPT 12 months
INTERNATIONAL MULTICENTRE DISCO REGISTRY [50]	All spontaneous coronary artery dissection	50.9 +/− 7.4	41.7 %	SCAD only	X	X	X	Included	Included	Included	Included	Included	Not reported	OCT/IVUS, 83%	X
SHERPA MAGIC STUDY [51]	All coronary presentations at clinicians' discretion	56.9 +/− 9	78%	All lesions	2.8–3.8	<24	X	Included	Excluded	Excluded	Included	X	73%	OCT/IVUS, 56%	DAPT 12 months
IT MASTERS REGISTRY [52]	Stable angina, NSTEMI, silent ischaemia.	59 +/− 6	83%	De Novo	X	<25	X	Included	Included	Included	Included	Included	61%	X	X
BIOMAG-1 [53,54]	Symptomatic coronary disease excluded STEMI	61 +/− 9	77.8 %	De Novo	2.5–4.2	<28	50–99%	Excluded	Excluded	Excluded	Excluded	Excluded	76.9%	OCT/IVUS, 97%	DAPT 12 months

SD, standard deviation; LVEF, left ventricular ejection fraction; X, not reported or not available; OCT, optical coherence tomography; IVUS, intravascular ultrasound; DAPT, dual antiplatelet therapy.

Table S4. Clinical Follow-Up Rates from Included Magnesium-Based Bioresorbable Stent Trials.

Study Name	Follow-Up			
	12 Months	24 Months	36 Months	60 Months
PROGRESS-AMS [22]	100%			
BIOSOLVE-I [23,24]	93.5%			
BIOSOLVE-II [25–28]	99%	95%	91%	60%
BIOSOLVE-III [29,30]	95.6%	94%	89.7%	
BEST-MAG [31]	94%			
MAGMARIS ACS REGISTRY [32,33]	79%	99%		
MAGSTEMI [34,35]	100%		100%	
Ghafari et al. [36]	100%			
Carlier et al. [37]	100%			
BIOSOLVE-IV [38,39]	97.6%	96.8%		
CardioHULA REGISTRY [40]	100%			
Franze et al. [41]	100%			
Guitierrez-Barrios et al. [42]	100%			
BIFSORB Pilot II [43]	63.6%			
PRAGUE-22 [44]	100%			
Fallesen et al. [45]	84%			
MULTICENTRE ITALIAN REGISTRY [46]	97.6%	92.8%		
Al Nooryani et al. [47]			98%	
Bossard et al. [48]	95%	94%	89%	86%
Truong et al. [49]	100%			
INTERNATIONAL MULTICENTRE DISCO REGISTRY [50]	100%			
SHERPA MAGIC STUDY [51]	100%			
IT MASTERS REGISTRY [52]	53%			
BIOMAG-1 [53,54]	98.3%	99.1%	96.6%	

Equation S1. Equation used to convert area to diameter.

$$\text{Diameter (mm)} = 2\sqrt{\frac{\text{Area (mm}^2\text{)}}{\pi}}$$

Table S5. Optical coherence tomography and quantitative angiography data from Fallesen et al. on late lumen loss.

	**OCT (*N* = 31)**	**QCA (*N* = 32)**
Minimal lumen area post procedure	7.0 ± 1.7 mm^2	6.8 ± 1.6 mm^2
Minimal lumen area at follow-up	4.7 ± 1.7 mm^2	5.4 ± 1.9 mm^2
Adjusted LLL	0.54 ± 1.7 mm	0.32 ± 1.76 mm
